# Residue class biases in unrestricted partitions, partitions into distinct parts, and overpartitions

**DOI:** 10.1007/s40687-025-00502-0

**Published:** 2025-02-21

**Authors:** Michael J. Schlosser, Nian Hong Zhou

**Affiliations:** 1 Fakultät für Mathematik, Universität Wien, Oskar-Morgenstern-Platz 1, 1090 Vienna, Austria; 2 School of Mathematics and Statistics, The Center for Applied Mathematics of Guangxi, Guangxi Normal University, Guilin, 541004 Guangxi People’s Republic of China

**Keywords:** Residue class biases, Unrestricted partitions, Distinct partitions, Overpartitions, Asymptotics, Primary 05A17, Secondary 11P81

## Abstract

We prove specific biases in the number of occurrences of parts belonging to two different residue classes *a* and *b*, modulo a fixed nonnegative integer *m*, for the sets of unrestricted partitions, partitions into distinct parts, and overpartitions. These biases follow from inequalities for residue-weighted partition functions for the respective sets of partitions. We also establish asymptotic formulas for the numbers of partitions of size *n* that belong to these sets of partitions and have a symmetric residue class bias (i.e., for $1\le a<m/2$ and $b=m-a$), as *n* tends to infinity.

## Introduction

Let ${\mathbb {N}}$ and ${\mathbb {N}}_0$ denote the sets of positive and nonnegative integers, respectively. Throughout this paper, we assume *q* to be a fixed complex number satisfying $0<|q|<1$. For any indeterminant *a* and complex number *c*, let the *q*-*shifted factorial* (cf. [12]) be defined by

$$\begin{aligned} (a;q)_\infty :=\prod _{j\ge 0}(1-aq^j),\quad \text {and}\quad (a;q)_c:=\frac{(a;q)_\infty }{(aq^c;q)_\infty }. \end{aligned}$$

Products of *q*-shifted factorials are compactly denoted as

$$\begin{aligned} (a_1,\ldots , a_m;q)_c:=\prod _{1\le j\le m}(a_j;q)_c \end{aligned}$$

for $$m\in {\mathbb {N}}$$ and $$c\in {{\mathbb {C}}}\cup \{\infty \}$$.

A partition $\lambda$ of a positive integer *n* (cf. [2]) is a nonincreasing sequence of positive integers (called the parts), $\lambda =(\lambda _1, \lambda _2,\ldots ,\lambda _l)$, such that $\lambda _1+\lambda _2+\cdots +\lambda _l=n$. The integer $l=:\ell (\lambda )$ is called the number of parts of $\lambda$. If $\lambda$ is a partition of *n* then we write the size of $\lambda$ as $|\lambda |=n$. We also define that $\ell (\varnothing )=|\varnothing | = 0$ for the empty partition, $\varnothing$, of 0. Let ${\mathcal {P}}(n)$ and ${{\mathcal {D}}}(n)$ denote the sets of all partitions and all partitions with distinct parts (which we shall call “distinct partitions” for short), of *n*, respectively. Let ${\mathcal {P}}=\bigcup _{n\ge 0}{\mathcal {P}}(n)$ and ${{\mathcal {D}}}=\bigcup _{n\ge 0}{{\mathcal {D}}}(n)$ denote the sets of all partitions and distinct partitions, respectively. Let $a,b, m\in {\mathbb {N}}$ with $1\le a<b\le m$. We define $\ell _{a,m}(\lambda )$ to be the number of parts of $\lambda$ congruent to *a* modulo *m*, and define $\ell _{a,m}(\lambda ,\mu ):=\ell _{a,m}(\lambda )+\ell _{a,m}(\mu )$ for each pair $(\lambda , \mu )\in {\mathcal {P}}\times {{\mathcal {D}}}$.

In this paper, we consider the following weighted partition function, defined as

1.1 $$\begin{aligned} p_n(x,y)=\sum _{\begin{array}{c} |\lambda |+|\mu |= n\\ (\lambda , \mu )\in {\mathcal {P}}\times {{\mathcal {D}}} \end{array}}x^{\ell (\lambda )}y^{\ell (\mu )}. \end{aligned}$$

where *x* and *y* are nonnegative real numbers, and $n\in {\mathbb {N}}_0$. It is clear that $p(n)=p_n(1,0)$, $q(n)=p_n(0,1)$ and ${\overline{p}}(n)=p_n(1,1)$ are the unrestricted (or ordinary) partition function, distinct partition function, and overpartition function, respectively. (Overpartitions were first explicitly considered by Brenti in [6, Section 3] who called them “dotted partitions.” A thorough independent study of these objects, involving bijections, generating functions and connections to Bailey chains, was initiated by Corteel and Lovejoy [8] who gave them the name “overpartitions,” which we follow.) We will focus on the residue-weighted biases partition functions which we define by

1.2 $$\begin{aligned} p_{n}(a,b,m; x,y):=\sum _{\begin{array}{c} |\lambda |+|\mu | =n\\ (\lambda , \mu )\in {\mathcal {P}}\times {{\mathcal {D}}}\\ \ell _{a,m}(\lambda ,\mu )> \ell _{b,m}(\lambda ,\mu ) \end{array}} x^{\ell (\lambda )}y^{\ell (\mu )}. \end{aligned}$$

From standard arguments of partition theory (cf. [2]), we have the following generating function for the statistics $\ell (\lambda )$ on ${\mathcal {P}}$, $\ell (\mu )$ on ${{\mathcal {D}}}$, $\ell _{a,m}(\lambda ,\mu ), \ell _{b,m}(\lambda ,\mu )$ and $|\lambda |+|\mu |$ on ${\mathcal {P}}\times {{\mathcal {D}}}$:

1.3 $$\begin{aligned}&\sum _{(\lambda , \mu )\in {\mathcal {P}}\times {{\mathcal {D}}}} u^{\ell _{a,m}(\lambda ,\mu )}v^{\ell _{b,m}(\lambda ,\mu )} x^{\ell (\lambda )}y^{\ell (\mu )}q^{|\lambda |+|\mu |}\nonumber \\&=\frac{(-yq;q)_\infty ( -uyq^a,-vyq^b,xq^a,xq^b;q^m)_\infty }{(xq;q)_\infty (-yq^a,-yq^b,uxq^a,vxq^b;q^m)_\infty }. \end{aligned}$$

Special cases of (1.3) are of interest. The case $(x,y)=(1,0)$, that is

$$\begin{aligned} \sum _{\lambda \in {\mathcal {P}}}u^{\ell _{a,m}(\lambda )} v^{\ell _{b,m}(\lambda )}q^{|\lambda |}= \frac{(q^a,q^b;q^m)_\infty }{(q;q)_\infty (uq^a,vq^b;q^m)_\infty }, \end{aligned}$$

was studied by Chern who in [7, Theorem 1.3] proved that

$$\begin{aligned} p_{n}(a,b,m; 1,0)\ge p_{n}(b,a,m; 1,0) \end{aligned}$$

holds for all $n\ge 0$ and $1\le a<b\le m$. Already earlier, Kim–Kim–Lovejoy [17] proved a phenomenon of parity bias for integer partitions, namely: the number of partitions of *n* ($n>0$, $n\ne 2$) with more odd parts than even parts is greater than the number of partitions of *n* ($n>0$, $n\ne 2$) with more even parts than odd parts. In our notation, they proved

1.4 $$\begin{aligned} p_{n}(1,2,2; 1,0)\ge p_{n}(2,1,2; 1,0) \end{aligned}$$

for all $n\ge 0$, where the inequality is strict except for $n\in \{0, 2\}$. Similar phenomena were shown by Kim–Kim [16] and include the following inequalities:

$$\begin{aligned} p_{n}(1,m,m; 1,0)\ge p_{n}(m,1,m; 1,0)\quad \text {and}\quad p_{n}(1,m-1,m; 1,0)\ge p_{n}(m-1,1,m; 1,0), \end{aligned}$$

for all integers $m\ge 2$ and $n\ge 0$.

While the inequality (1.4) of partitions holds for all $n\ge 0$, the same inequality holds only from a certain integer on when restricting to the set of distinct partitions. In fact, Kim–Kim–Lovejoy [17] conjectured (in our notation) that

$$\begin{aligned} p_{n}(1,2,2; 0,1)> p_{n}(2,1,2; 0,1) \end{aligned}$$

for all $n>19$ (only); this has been proved by Banerjee–Bhattacharjee–Dastidar–Mahanta–Saikia [4] by using a combinatorial approach.

In this paper, we are interested in the biases of $p_{n}(a,b,m; x,y)$ in terms of different residue classes *a* and *b* of a fixed modulus *m*. Our main results are the following three Theorems 1.1–1.3.

### Theorem 1.1

Let *a*, *b*, *m* be any integers such that $1\le a<b\le m$. For any $x\ge 1$, $y\ge 0$ and $n\ge 0$, we have

$$\begin{aligned} p_{n}(a,b,m; x,y)\ge p_{n}(b,a,m; x,y). \end{aligned}$$

We point out that the case $(x,y)=(1,1)$ of Theorem 1.1, that is the case related to the generating function

$$\begin{aligned} \sum _{(\lambda , \mu )\in {\mathcal {P}}\times {{\mathcal {D}}}} u^{\ell _{a,m}(\lambda ,\mu )}v^{\ell _{b,m}(\lambda ,\mu )} q^{|\lambda |+|\mu |}= \frac{(-q;q)_\infty (-uq^a,-vq^b,q^a,q^b;q^m)_\infty }{(q;q)_\infty (-q^a,-q^b,uq^a,vq^b;q^m)_\infty }, \end{aligned}$$

is the overpartitions analogue of the aforementioned result by Chern [7]. We also establish some new results for the bias of distinct partitions, belonging to the case $(x,y)=(0,1)$. In particular, for any $x\ge 0$ and $y=1$, we have the following theorem.

### Theorem 1.2

Let $x\ge 0$ and *a*, *b*, *m* be integers such that $1\le a<b\le m$. Assume that there exists a positive integer *k* with $k\mid (b-a)$ such that neither of the congruences

$$\begin{aligned} 2^{h}k\equiv a\pmod m\;\;\text {and}\;\; 2^{h}k\equiv b\pmod m, \end{aligned}$$

possesses a solution for any $h\in {\mathbb {N}}$. Then for all integers $n\ge 0$, we have

$$\begin{aligned} p_{n}(a,b,m; x,1)\ge p_{n}(b,a,m; x,1). \end{aligned}$$

### Remark 1.1

Let *a*, *b*, *m* be integers such that $1\le a<b\le m$. It is not difficult to check that the following triples (*a*, *b*, *m*) meet the conditions in Theorem 1.2.

1. Any even integer *m* and odd integers *a*, *b*. (In this case, $k=2$ guarantees the nonexistence of solutions for any of the two congruences.)
2. Any integers *m*, *a*, *b* such that $\gcd (a,b, m)$ has an odd factor $\ge 3$. (In this case, one can take $k=1$.)

We finally prove the following asymptotic formulas for weighted partitions with biases of *symmetric* residue classes (by which we mean that in the biases of $p_n(a,b,m;x,y)$ the two residue classes *a* and *b* satisfy the symmetry $b=m-a$ where $1\le a<m/2$). Below, when writing *a*/2*m* we mean *a*/(2*m*), and other fractions in one line notation (with denominators consisting of products) are to be similarly interpreted in the remainder of this paper.

### Theorem 1.3

For any given $1\le a< m/2$ and $(x,y)\in \{(1,0),(0,1),(1,1)\}$, we have

$$\begin{aligned} p_{n}(a,m-a,m; x,y)\sim c_{a,m}(x)p_n(x,y), \end{aligned}$$

as $$n\rightarrow +\infty $$, where

$$\begin{aligned} \left( c_{a,m}(0), c_{a,m}(1)\right) = \left( \frac{1}{2},~\frac{\psi \left( 1/2+a/2m\right) - \psi \left( {a}/{2m}\right) }{2\pi \csc (a\pi /m)}\right) , \end{aligned}$$

and where $\psi (x)=\Gamma '(x)/\Gamma (x)$ is the digamma function.

The proof of Theorem 1.3 employs a residue class analogue of Ingham’s Tauberian theorem [14, Theorem 1], which, to the best of the authors’ knowledge, has not appeared before in the literature. We state it as the following theorem and believe that it is of independent interest. We give its proof in Sect. 3.3.

### Theorem 1.4

Let $f(q)=\sum _{n\ge 0}c_nq^n$ be a power series whose coefficients $c_n$ are nonnegative. Suppose that for some positive integer *m* and any integer $1\le h<m$, one has

1. $c_{n+m}\ge c_n$ for all $n\ge 0$, and
2. there exist constants $\gamma \in {\mathbb {R}}$ and $\alpha , \beta ,\rho \in {\mathbb {R}}_+$ such that
  $$\begin{aligned} f(e^{-z})\sim \alpha z^{\gamma }\exp \left( \beta z^{-\rho }/\rho \right) \;\;\text {and}\;\; f(e^{-z}e^{2\pi \textrm{i}h/m})=o\left( f(e^{-z})\right) , \end{aligned}$$
  when $z\rightarrow 0$ in every fixed Stolz angle $|\arg (z)|\le \Delta$ $(0<\Delta <\pi /2)$.

Then, as $$n\rightarrow \infty $$,

$$\begin{aligned} c_n\sim \frac{\alpha \beta ^{\frac{1+2\gamma }{2(1+\rho )}}}{\sqrt{2\pi (1+\rho )}}n^{-\frac{1+2\gamma }{2(1+\rho )}-\frac{1}{2}} \exp \left( (1+1/\rho )\beta ^{1/(1+\rho )} n^{\rho /(1+\rho )}\right) . \end{aligned}$$

The rest of the paper is organized as follows. In Sect. 2, we give the proof of Theorems 1.1 and 1.2. This is achieved with the help of some *q*-series transformations and a new overpartitions analogue of a theorem of Andrews [1, Theorem 3]. In Sect. 3, we first provide the proof of our residue class analogue of Ingham’s Tauberian theorem, i.e., of Theorem 1.4. Then, we turn to partition functions with bias of symmetric residue classes and establish their generating functions and asymptotics. We close in Sect. 4 with some remarks on residue class biases in distinct partitions.

## The proofs of Theorems 1.1 and 1.2

In this section, we provide proofs of Theorems 1.1 and 1.2. To study $p_{n}(a,b,m; x,y)$, we first derive its generating function. Using Cauchy’s *q*-binomial theorem (cf. [12, Appendix (II.3)]):

$$\begin{aligned} \frac{(\alpha t;q)_\infty }{(t;q)_\infty } =\sum _{n\ge 0}\frac{(\alpha ;q)_nt^n}{(q;q)_n}, \end{aligned}$$

valid for $|t|<1$, we have

$$\begin{aligned} \frac{(-uyq^a,-vyq^b;q^m)_\infty }{(uxq^a,vxq^b;q^m)_\infty } =\sum _{n_1\ge 0}\frac{(-y/x;q^m)_{n_1}(xuq^{a})^{n_1}}{(q^m;q^m)_{n_1}} \sum _{n\ge 0}\frac{(-y/x;q^m)_{n}(xvq^{b})^{n}}{(q^m;q^m)_{n}}. \end{aligned}$$

Therefore, by the definition (1.2) for $p_{n}(a,b,m; x,y)$ and (1.3), we obtain

2.1 $$\begin{aligned} \sum _{n\ge 0}p_{n}(a,b,m; x,y)q^n&=\frac{(-yq;q)_\infty (xq^a,xq^b;q^m)_\infty }{(xq;q)_\infty (-yq^a,-yq^b;q^m)_\infty }\nonumber \\&\quad \times \sum _{\begin{array}{c} n_1,n\ge 0\\ n_1> n \end{array}} \frac{x^{n_1}(-y/x;q^m)_{n_1}x^{n}(-y/x;q^m)_{n}q^{an_1+bn}}{(q^m;q^m)_{n_1}(q^m;q^m)_{n}}. \end{aligned}$$

Writing $n_1=k+1+n$ in (2.1), and using the simple identity $(\alpha ; q)_{n+1+k}=(1-\alpha )(\alpha q;q)_{n+k}$, we get

2.2 $$\begin{aligned} (1-q^m)\sum _{n\ge 0}p_{n}(a,b,m; x,y)q^n =\frac{(-yq;q)_\infty (xq^a,xq^b;q^m)_\infty }{(xq;q)_\infty (-yq^a,-yq^b;q^m)_\infty }(x+y)q^a&\nonumber \\ \times \sum _{n,k\ge 0} \frac{(-q^my/x;q^m)_{n+k}(-y/x;q^m)_n x^{2n+k}q^{(a+b)n+ka}}{(q^{2m};q^m)_{n+k}(q^m;q^m)_{n}}&. \end{aligned}$$

Equation (2.2) immediately yields the following monotonicity result, which we will use for deriving asymptotic formulas for $p_{n}(a,m-a,m; x,y)$ in Sect. 3.

### Lemma 2.1

For any $x,y\ge 0$ and $n\ge 0$, we have

$$\begin{aligned} p_{n+m}(a,b,m; x,y)\ge p_{n}(a,b,m; x,y). \end{aligned}$$

Equation (2.2) also implies that

$$\begin{aligned}&\sum _{n\ge 0} \left( p_{n}(a,b,m; x,y)-p_{n}(b,a,m; x,y)\right) q^n\nonumber \\&=\frac{1}{1-q^m}(1-q^{b-a})\frac{(-yq;q)_\infty (xq^a,xq^b;q^m)_\infty }{(xq;q)_\infty (-yq^a,-yq^b;q^m)_\infty }(x+y)q^a\nonumber \\&\quad \times \sum _{n,k\ge 0}\frac{(1-q^{(k+1)(b-a)})(-q^my/x;q^m)_{n+k} (-y/x;q^m)_n x^{2n+k}q^{(a+b)n+ak}}{(1-q^{b-a})(q^{2m};q^m)_{n+k}(q^m;q^m)_{n}}. \end{aligned}$$

Note that the *q*-series expansion of the above infinite double sum has nonnegative coefficients, with constant term equal to 1, provided that $1\le a<b\le m$ and $x,y\ge 0$, because of

$$\begin{aligned} \frac{1-q^{(k+1)(b-a)}}{1-q^{b-a}}=\sum _{0\le j\le k}q^{j(b-a)}. \end{aligned}$$

This immediately implies the following lemma.

### Lemma 2.2

Let $1\le a<b\le m$ and $x,y\ge 0$ with $x+y> 0$. Define

$$\begin{aligned} f_{a,b,m,x,y}(q)=(1-q^{b-a})\frac{(-yq;q)_\infty }{(xq;q)_\infty } \frac{(xq^a,xq^b;q^m)_\infty }{(-yq^a,-yq^b;q^m)_\infty }. \end{aligned}$$

Then,

$$\begin{aligned} p_{n}(a,b,m; x,y)\ge p_{n}(b,a,m; x,y)\ge 0 \end{aligned}$$

for all integers $n\ge 0$, provided that all the Taylor coefficients of $f_{a,b,m,x,y}(q)$ about $q=0$ are nonnegative.

Lemma 2.2 will be used to give the proof of Theorem 1.1 in the cases $(a,b)\ne (1,2)$, and the proof of Theorem 1.2. To achieve that we will need the following proposition.

### Proposition 2.3

For $x\ge 1, y\ge 0$ and $(a,b)\ne (1,2)$, all the Taylor coefficients of $f_{a,b,m,x,y}(q)$ about *q* are nonnegative.

### Proof

Note that for $1<a<b\le m$, we have

$$\begin{aligned} f_{a,b,m,x,y}(q)=\frac{1-q^{b-a}}{1-xq}\frac{(-yq;q^m)_\infty }{(xq^{m+1};q^m)_\infty }\prod _{\begin{array}{c} 1\le j\le m\\ j\not \in \{1,a,b\} \end{array}} \frac{(-yq^j;q^m)_\infty }{(xq^j;q^m)_{\infty }} \end{aligned}$$

and

$$\begin{aligned} \frac{1-q^{b-a}}{1-x q}= \left( 1+\sum _{j\ge 1}(x^{j}-x^{j-1})q^j\right) \sum _{0\le h<b-a}q^{h}; \end{aligned}$$

and for $a=1$ with $b>2$, we have

$$\begin{aligned} f_{a,b,m,x,y}(q)=\frac{1-q^{b-1}}{1-x q^{b-1}} \frac{(-yq^{b-1};q^m)_\infty }{(xq^{m+b-1};q^m)_\infty } \prod _{\begin{array}{c} 1\le j\le m\\ j\not \in \{1,b-1, b\} \end{array}} \frac{(-yq^j;q^m)_\infty }{(xq^j;q^m)_{\infty }} \end{aligned}$$

and

$$\begin{aligned} \frac{1-q^{b-1}}{1-x q^{b-1}}=1+\sum _{j\ge 1}(x^{j}-x^{j-1})q^{(b-1)j}. \end{aligned}$$

Since $x\ge 1$, it follows that all the Taylor coefficients of $f_{a,b,m,x,y}(q)$ about $q=0$ are nonnegative. This completes the proof of the proposition. $\square$

To prove Theorem 1.1 for the remaining case $(a,b)=(1,2)$, we use another method. For any $1\le a<b\le m$, by writing $n_1=k+1+n$ in (2.1) and using the elementary identity

$$\begin{aligned} (\alpha ; q)_{n+1+k}=(\alpha ;q)_n(\alpha q^{n};q)_{k+1}, \end{aligned}$$

we find that

$$\begin{aligned} \sum _{n\ge 0}p_{n}(a,b,m; x,y)q^n&= \frac{(-yq;q)_\infty (xq^a,xq^b;q^m)_\infty }{(xq;q)_\infty (-yq^a,-yq^b;q^m)_\infty }\\&\quad \times \sum _{n\ge 0}\frac{(-y/x;q^m)_{n}^2 (x+yq^{mn})x^{2n}q^{(a+b)n}}{(q^m;q^m)_{n}^2}\\&\quad \times \sum _{k\ge 0}\frac{(-q^{m(n+1)}{y}/{x};q^m)_{k} x^kq^{a(k+1)}}{(q^{m(n+1)};q^m)_{k+1}}. \end{aligned}$$

Thus,

2.3 $$\begin{aligned}&\sum _{n\ge 0}\left( p_{n}(a,b,m; x,y)-p_{n}(b,a,m; x,y)\right) q^n\nonumber \\&=\frac{(-yq;q)_\infty (xq^a,xq^b;q^m)_\infty }{(xq;q)_\infty (-yq^a,-yq^b;q^m)_\infty } \sum _{n\ge 0}\frac{(-y/x;q^m)_{n}^2 (x+yq^{mn})x^{2n}q^{(a+b)n}}{(q^m;q^m)_{n}^2}\nonumber \\&\quad \times \left( \sum _{k\ge 0}\frac{(-q^{m(n+1)}{y}/{x};q^m)_{k} x^kq^{a(k+1)}}{(q^{m(n+1)};q^m)_{k+1}}- \sum _{k\ge 0}\frac{(-q^{m(n+1)}{y}/{x};q^m)_{k}x^kq^{b(k+1)}}{(q^{m(n+1)};q^m)_{k+1}}\right) . \end{aligned}$$

From Equation (2.3), it is obvious that to prove $p_{n}(1,2,m; x,y)\ge p_{n}(2,1,m; x,y)$, it suffices to prove the following theorem, which restricts to such pairs (*a*, *b*) where *b* is a multiple of *a* (and thus can be replaced by *ab*), which is more than we need for the case $(a,b)=(1,2)$.

### Theorem 2.4

Let $x\ge 1$, $y\ge 0$ and $a,b\in {\mathbb {N}}$. For all integers $m,s\ge 1$, the *q*-series expansion of

$$\begin{aligned} \sum _{k\ge 0}\frac{(-yq^{s}/x;q^m)_{k}x^{k}q^{a(k+1)}}{(q^{s};q^m)_{k+1}} -\sum _{k\ge 0}\frac{(-yq^{s}/x;q^m)_{k}x^{k}q^{ab(k+1)}}{(q^{s};q^m)_{k+1}} \end{aligned}$$

has nonnegative coefficients.

We note that the $(x,y,a,b)=(1,0,1,2)$ special case of Theorem 2.4 was conjectured by Chern [7, Conjecture 4.2] and subsequently proved by Binner [5, Section 2], which we state as the following corollary:

### Corollary 2.5

For all integers $m,s\ge 1$, the *q*-series expansion of

$$\begin{aligned} \sum _{k\ge 0}\frac{q^k(1-q^k)}{(q^{s};q^m)_k} \end{aligned}$$

has nonnegative coefficients.

### Proof of Theorem 2.4

Using the Heine transformation [12, Appendix (III.2)] (valid for $|z|<1$ and $|\gamma /\beta |<1$)

$$\begin{aligned} \sum _{n\ge 0}\frac{(\alpha ,\beta ;q)_n}{(\gamma ,q;q)_n}z^n= \frac{(\gamma /\beta ,\beta z;q)_\infty }{(\gamma ,z;q)_\infty } \sum _{n\ge 0}\frac{(\alpha \beta z/\gamma ,\beta ;q)_n}{(\beta z,q;q)_n}(\gamma /\beta )^n, \end{aligned}$$

and making the substitution $\beta \mapsto q, \gamma \mapsto \gamma q$, one readily obtains

$$\begin{aligned} \sum _{n\ge 0}\frac{(\alpha ;q)_n}{(\gamma ;q)_{n+1}}z^n= \sum _{n\ge 0}\frac{(\alpha z/\gamma ;q)_n}{(z;q)_{n+1}}\gamma ^n \end{aligned}$$

(which can be referred to as Fine’s transformation [10, Equation (6.3)], as Fine thoroughly studied this type of series in his monograph on basic hypergeometric series), valid for $|z|<1$ and $|\gamma |<1$. Thus,

$$\begin{aligned}&\sum _{k\ge 0}\frac{(-yq^{s}/x;q^m)_{k}(q^a(xq^{a})^k- q^{ab}(xq^{ab})^k)}{(q^{s};q^m)_{k+1}}\\  &= \sum _{k\ge 0}\left( \frac{q^a(-yq^a;q^m)_{k}}{(xq^{a};q^m)_{k+1}}- \frac{q^{ab}(-yq^{ab};q^m)_{k}}{(xq^{ab};q^m)_{k+1}}\right) q^{sk}. \end{aligned}$$

Note that, for any integer $k \ge 0$,

$$\begin{aligned}&\frac{(-yq^a;q^m)_{k}xq^a}{(xq^a;q^m)_{k+1}}- \frac{(-yq^{ab};q^m)_{k}xq^{ab}}{(xq^{ab};q^m)_{k+1}}\\  &= \sum _{h\ge 1}\left( \frac{(-yq^a;q^m)_{k}(xq^a)^{h}}{(xq^a;q^m)_{k}} -\frac{(-yq^{ab};q^m)_{k}(xq^{ab})^{h}}{(xq^{ab};q^m)_{k}}\right) q^{(h-1)km}. \end{aligned}$$

The proof of Theorem 2.4 now is an immediate consequence of Theorem 2.6, which we provide subsequently. $\square$

The following result is an overpartition analogue of a result by Andrews [1, Theorem 3].

### Theorem 2.6

Let $(a_j)_{j\ge 0},(b_j)_{j\ge 0}$ be two increasing sequences of positive integers with $b_0\equiv 0 \pmod {a_0}$, $b_0>a_0$ and $b_j-a_j\equiv 0\pmod {a_0}$ for each $j\ge 1$. For any $n\in {\mathbb {N}}_0$, $h\in {\mathbb {N}}_0$ and $x\ge 1, y\ge 0$, the *q*-series expansion of

$$\begin{aligned} (xq^{a_0})^h\prod _{0\le j\le n}\frac{1+yq^{a_j}}{1-xq^{a_j}} -(xq^{b_0})^h\prod _{0\le j\le n}\frac{1+yq^{b_j}}{1-xq^{b_j}} \end{aligned}$$

has nonnegative coefficients.

### Proof

For $n=0$, the *q*-series expansion of

$$\begin{aligned}&\frac{1+yq^{a_0}}{1-xq^{a_0}} (xq^{a_0})^h- \frac{1+yq^{b_0}}{1-xq^{b_0}} (xq^{b_0})^h\\&=(xq^{a_0})^h-(xq^{b_0})^h+(1+y/x) \sum _{k>h}((xq^{a_0})^{k}-(xq^{b_0})^k)\\&=x^hq^{a_0h}+(1+y/x)\sum _{h<k< b_0h/a_0}x^{k}q^{a_0k} +yx^{h-1}q^{b_0h}\\&\quad +(1+y/x)\sum _{\begin{array}{c} k\ge h\\ b_0\not \mid a_0k \end{array}}x^kq^{a_0k} +(1+y/x)\sum _{k\ge h}(x^{b_0k/a_0}-x^{k})q^{b_0k} \end{aligned}$$

has nonnegative coefficients (since $x\ge 1$). Note that

2.4 $$\begin{aligned}&(xq^{a_0})^h\prod _{0\le j\le n}\frac{1+yq^{a_j}}{1-xq^{a_j}}-(xq^{b_0})^h\prod _{0\le j\le n} \frac{1+yq^{b_j}}{1-xq^{b_j}}\nonumber \\&=\left( \frac{1+y q^{a_n}}{1-xq^{a_n}}- \frac{1+y q^{b_n}}{1-xq^{b_n}}\right) (xq^{a_0})^h \prod _{0\le j< n}\frac{1+yq^{a_j}}{1-xq^{a_j}}\nonumber \\&\quad +\frac{1+y q^{b_n}}{1-xq^{b_n}} \left( (xq^{a_0})^h\prod _{0\le j\le n-1} \frac{1+yq^{a_j}}{1-xq^{a_j}} -(xq^{b_0})^h\prod _{0\le j\le n-1} \frac{1+yq^{b_j}}{1-xq^{b_j}}\right) , \end{aligned}$$

and

2.5 $$\begin{aligned}&\left( \frac{1+y q^{a_n}}{1-xq^{a_n}}-\frac{1+y q^{b_n}}{1-xq^{b_n}}\right) \prod _{0\le j< n}\frac{1+yq^{a_j}}{1-xq^{a_j}}\nonumber \\&=\frac{ 1-q^{b_n-a_n}}{1-xq^{a_0}}\frac{q^{a_n}(x+y)}{1-x q^{b_n}} \prod _{0\le j< n}\frac{1+yq^{a_j}}{1-xq^{a_{j+1}}}. \end{aligned}$$

Now, since the *q*-series expansion of the factor

$$\begin{aligned} \frac{1-q^{b_n-a_n}}{1-xq^{a_0}}= \left( 1+\sum _{j\ge 1}(x^{j}-x^{j-1})q^{a_0j}\right) \sum _{0\le j< (b_n-a_n)/a_0}q^{a_0 j} \end{aligned}$$

has nonnegative coefficients, we can conclude that the *q*-series expansion of (2.5) has nonnegative coefficients as well. Using (2.4), the proof is established by induction. $\square$

We are now ready to give the proof of Theorem 1.2.

### Proof of Theorem 1.2

Since both of the congruence equations

$$\begin{aligned} 2^{h+1}k\equiv a\pmod m\;\;\text {and}\;\; 2^{h+1}k\equiv b\pmod m \end{aligned}$$

possess no solution for $h\in {\mathbb {N}}_0$, we have that both of the equations

$$\begin{aligned} 2^{h_1}k=m\ell _1+a\;\; \text {and}\;\; 2^{h_2}k=m\ell _2+b \end{aligned}$$

possess no nonnegative integer solutions $(h_1,\ell _1)$ and $(h_2, \ell _2)$. This yields

$$\begin{aligned} \{2^hk: h\in {\mathbb {N}}_0\}\subseteq \{j\in {\mathbb {N}}: j\not \equiv a, b\pmod m\}. \end{aligned}$$

On the other hand, since $b>a$ and $k\mid (b-a)$, it is clear that the *q*-series expansion of $(1-q^{b-a})/(1-q^{k})$ has nonnegative coefficients. Thus,

$$\begin{aligned} f_{a,b,m,x,1}(q)=\frac{(1-q^{k})(-q;q)_\infty }{(-q^a,-q^{b};q^m)_\infty }\cdot \frac{1-q^{b-a}}{1-q^k} \prod _{\begin{array}{c} 1\le j\le m\\ j\not \in \{a,b\} \end{array}}\frac{1}{(xq^j;q^m)_{\infty }} \end{aligned}$$

has nonnegative coefficients in its *q*-expansion too, since $x\ge 0$ and the *q*-series expansion of

$$\begin{aligned} \frac{(1-q^{k})(-q;q)_\infty }{(-q^a,-q^{b};q^m)_\infty } =(1-q^{k})\prod _{h\ge 0}(1+q^{2^hk}) \prod _{\begin{array}{c} j\ge 1\\ j\not \equiv a,b\pmod m\\ j\ne 2^{h}k, h\in {\mathbb {N}}_0 \end{array}} \left( 1+q^j\right)&\\= \prod _{\begin{array}{c} j\ge 1\\ j\not \equiv a,b\pmod m\\ j\ne 2^{h}k, h\in {\mathbb {N}}_0 \end{array}} \left( 1+q^j\right)&\end{aligned}$$

has nonnegative coefficients. Therefore, by using Lemma 2.2 and Proposition 2.3 we get

$$\begin{aligned} p_{n}(a,b,m;x,1)-p_{n}(b,a,m;x,1)\ge 0, \end{aligned}$$

which completes the proof of Theorem 1.2. $\square$

## Weighted partitions with bias of symmetric residue classes

Recall that $p_n(a,b,m;x,y)$ is defined by (1.2). Let $(x,y)\in S$ where $S=\{(0,1), (1,0), (1,1)\}$, and let *a*, *m* be positive integers such that $1\le a<m$ and $m\ne 2a$. We write

$$\begin{aligned} G_{xy}(a,m;q)=\sum _{n\ge 0}p_n(a,m-a,m;x,y)q^n \end{aligned}$$

for the generating function of weighted partitions with bias of *symmetric* residue classes. The first objective of this section, rather than just using (2.1), is to derive forms for the generating functions $G_{xy}(a,m;q)$, where $(x,y)\in S$, which will be convenient for establishing asymptotic formulas for $p_n(a,m-a,m;x,y)$, where $(x,y)\in S$.

### Generating functions for weighted partitions with bias of symmetric residue classes

To obtain the generating functions for $G_{xy}(a,m;q)$ with $(x,y) \in S$, we require the following identities:

3.1 $$\begin{aligned} (-\zeta , -q/\zeta , q;q)_\infty = \sum _{n\in {\mathbb {Z}}}q^{\frac{n(n-1)}{2}}\zeta ^n, \end{aligned}$$

3.2 $$\begin{aligned} \frac{(q;q)_\infty ^2}{(\zeta , q/\zeta ;q)_\infty }= \sum _{n\in {\mathbb {Z}}} \frac{(-1)^nq^{\frac{n(n+1)}{2}}}{1-\zeta q^n}, \end{aligned}$$

and

3.3 $$\begin{aligned} \frac{(-q/\zeta ,-\zeta ;q)_\infty }{(q/\zeta ,\zeta ;q)_\infty }= \frac{(-q;q)_\infty ^2}{(q;q)_\infty ^2}\left( 1+2\sum _{n\ge 1} \frac{\zeta ^n+(q/\zeta )^n}{1+q^n}\right) . \end{aligned}$$

The identity (3.1) is the well-known Jacobi triple product identity (cf. [12, Appendix (II.28)]). The identity (3.2) is the reciprocal of a theta function, which is expanded in terms of partial fractions by the Mittag–Leffler theorem. (For details, see Ramanujan’s lost notebook [3, Entry 3.2.1] or Garvan [11, Equation (7.15)].) The identity (3.3) is a special case of an identity originally due to Kronecker (cf. [18, pp. 70–71]) and can be easily derived from Ramanujan’s $_1\psi _1$ summation formula (cf. [12, Appendix (II.29)]).

#### Proof of identity (3.3)

Ramanujan’s $_{1}\psi _{1}$ summation formula is

$$\begin{aligned} \sum _{n\ge 0}\frac{(a;q)_n}{(b;q)_n}\zeta ^n+ \sum _{n\ge 1}\frac{(q/b;q)_{n}}{(q/a;q)_n}(b/a\zeta )^{n}= \frac{(b/a,q/a\zeta ,a\zeta ,q;q)_\infty }{(b,b/a\zeta ,q/a,\zeta ;q)_\infty }, \end{aligned}$$

provided $|\zeta |<1$ and $|b/a\zeta |<1$. Letting $b\mapsto aq$, this reduces to Kronecker’s bilateral summation

$$\begin{aligned} \sum _{n\ge 0}\frac{1-a}{1-aq^n}\zeta ^n+ \sum _{n\ge 1}\frac{1-1/a}{1-q^n/a}(q/\zeta )^{n}= \frac{(q,q/a\zeta ,a\zeta ,q;q)_\infty }{(aq,q/\zeta ,q/a,\zeta ;q)_\infty }. \end{aligned}$$

Letting $a\rightarrow -1$, we obtain after some rewriting (3.3). $\square$

We now give the generating functions for $G_{xy}(a,m;q)$ with $(x,y) \in S$.

#### Lemma 3.1

We have

$$\begin{aligned} G_{01}(a,m;q)=\frac{(q^2;q^2)_\infty }{(-q^a,-q^{m-a},q^m;q^m)_\infty (q;q)_\infty } \sum _{n\ge 1}q^{\frac{mn(n-1)}{2}+n a}, \\ G_{10}(a,m;q)=\frac{(q^a,q^{m-a};q^m)_\infty }{(q;q)_\infty (q^m;q^m)_\infty ^2}\sum _{n\ge 0}(-1)^{n} \frac{q^{m\frac{n(n+1)}{2}+mn+a}}{1-q^{mn+a}}, \end{aligned}$$

and

$$\begin{aligned} G_{11}(a,m;q)=2\frac{(q^2;q^2)_\infty (q^{2m};q^{2m})_\infty ^2}{(q;q)_\infty ^2(q^m;q^m)_\infty ^4}\frac{(q^a,q^{m-a};q^m)_\infty }{(-q^a,-q^{m-a};q^m)_\infty }\sum _{n\ge 1}\frac{q^{an}}{1+q^{mn}}. \end{aligned}$$

#### Proof

Recall that

$$\begin{aligned} G_{xy}(a,m;q)=\sum _{\begin{array}{c} (\lambda , \mu )\in {\mathcal {P}}\times {{\mathcal {D}}}\\ \ell _{a,m}(\lambda ,\mu )-\ell _{b,m}(\lambda ,\mu )>0 \end{array}} x^{\ell (\lambda )}y^{\ell (\mu )}q^{|\lambda |+|\mu |}, \end{aligned}$$

and, by the $u=t$, $v=t^{-1}$ case of (1.3),

$$\begin{aligned}&\sum _{(\lambda , \mu )\in {\mathcal {P}}\times {{\mathcal {D}}}} t^{\ell _{a,m}(\lambda ,\mu )-\ell _{b,m}(\lambda ,\mu )} x^{\ell (\lambda )}y^{\ell (\mu )}q^{|\lambda |+|\mu |}\\  &= \frac{(-yq;q)_\infty ( xq^a,xq^b;q^m)_\infty ( -tyq^a,-t^{-1}yq^b;q^m)_\infty }{(xq;q)_\infty (-yq^a,-yq^b;q^m)_\infty (txq^a,t^{-1}xq^b;q^m)_\infty }. \end{aligned}$$

Using identities (3.1)–(3.3), we have:

3.4 $$\begin{aligned} \sum _{\mu \in {{\mathcal {D}}}}t^{\ell _{a,m}(\mu )-\ell _{m-a,m}(\mu )}q^{|\mu |}= \frac{(-q;q)_\infty }{(-q^a,-q^{m-a},q^m;q^m)_\infty } \sum _{n\in {\mathbb {Z}}}q^{\frac{mn(n-1)}{2}+n a}t^{n}, \end{aligned}$$

3.5 $$\begin{aligned} \sum _{\mu \in {\mathcal {P}}}t^{\ell _{a,m}(\mu )-\ell _{m-a,m}(\mu )}q^{|\mu |}= \frac{(q^a,q^{m-a};q^m)_\infty }{(q;q)_\infty (q^m;q^m)_\infty ^2} \sum _{n\in {\mathbb {Z}}}\frac{(-1)^{n}q^{m\frac{n(n+1)}{2}}}{1-q^{mn+a}t}, \end{aligned}$$

and

3.6 $$\begin{aligned} \sum _{(\lambda , \mu )\in {\mathcal {P}}\times {{\mathcal {D}}}} t^{\ell _{a,m}(\lambda ,\mu )-\ell _{m-a,m}(\lambda ,\mu )}q^{|\lambda |+|\mu |}&=\frac{(q^2;q^2)_\infty (q^{2m};q^{2m})_\infty ^2(q^a,q^{m-a};q^m)_\infty }{(q;q)_\infty ^2(q^m;q^m)_\infty ^4(-q^a,-q^{m-a};q^m)_\infty }\nonumber \\&\quad \times \left( 1+2\sum _{n\ge 1}\frac{q^{an}t^{n}+q^{(m-a)n}t^{-n}}{1+q^{mn}}\right) . \end{aligned}$$

Therefore, by collecting the coefficients of $t^n$ for $n \in {\mathbb {N}}$ (and omitting the other coefficients where *n* is negative) in the above equations (3.4)–(3.6), we readily obtain the proof of the lemma. $\square$

### Asymptotics of the generating functions

In this subsection, we study the asymptotics of the *q*-series $G_{xy}(a,m;q)$, with $(x,y)\in S$, *q* nearing the *m*-th root of unity $\zeta _{m}^h:=e^{2\pi \textrm{i}h/m}$, where $m\in {\mathbb {N}}$ and $h\in {\mathbb {N}}_0$. In order to achieve this, we first need the following proposition which are special cases of results by Katsurada [15, Theorem 5, Corollary 1.1, Corollary 1.3].

#### Proposition 3.2

The following asymptotic formulas hold for $z\rightarrow 0$ in every fixed Stolz angle $|\arg (z)|\le \Delta$ $(0<\Delta <\pi /2)$.

1. For any $h\in {\mathbb {N}}_0$ and $m\in {\mathbb {N}}$ such that $\gcd (h,m)=1$, we have
  $$\begin{aligned} (e^{-z}\zeta _{m}^h;e^{-z}\zeta _{m}^h)_\infty = A_0(h,m)z^{-1/2}\exp \left( -\frac{\pi ^2}{6m^2z}\right) (1+O(z)), \end{aligned}$$
  where $A_0(h,m)$ is a constant. In particular, $A_0(1,1)=\sqrt{2\pi }$.
2. For any $\alpha >0$, we have
  $$\begin{aligned} (-e^{-\alpha z};e^{-z})_\infty = 2^{1/2-\alpha }\exp \left( \frac{\pi ^2}{12z}\right) (1+O(z)). \end{aligned}$$
3. For any $$\alpha , \mu \in (0,1)$$,
  $$\begin{aligned} (e^{-\alpha z}, e^{-(1-\alpha ) z};e^{-z})_{\infty }= 2\sin (\pi \alpha )\exp \left( -\frac{\pi ^2}{3z}\right) (1+O(z)), \end{aligned}$$
  and
  $$\begin{aligned}&(e^{2\pi \textrm{i}\mu }e^{-\alpha z},e^{2\pi \textrm{i}(1-\mu )}e^{-(1-\alpha ) z};e^{-z})_{\infty }\\&=e^{-2\pi \textrm{i}(1/2-\alpha )(1/2-\mu )}\exp \left( -\frac{2\pi ^2B_2(\mu )}{z}\right) (1+O(z)), \end{aligned}$$
  where $$B_2(\mu )=\mu ^2-\mu +1/6$$,^1^

The asymptotics of the infinite products in Lemma 3.1 will follow from Proposition 3.2. It remains to establish the asymptotics for the infinite sums in Lemma 3.1. In the following, let ${\textbf {1}}_{event}$ denote the indicator function, let $h\in {\mathbb {N}}_0$ and let $q=e^{-z/m}\zeta _m^h$. Let $\psi (u)=\Gamma '(u)/\Gamma (u)$ be the digamma function given by

$$\begin{aligned} \psi (u)=-\gamma +\sum _{n\ge 1}\left( \frac{1}{n}-\frac{1}{n+u}\right) . \end{aligned}$$

We prove the following lemmas.

#### Lemma 3.3

As $z\rightarrow 0$ in every fixed Stolz angle $|\arg (z)|\le \Delta$ $(0<\Delta <\pi /2)$, we have

3.7 $$\begin{aligned} \sum _{n\ge 1}q^{m\frac{n(n-1)}{2}+n a}= \frac{{\textbf {1}}_{m\mid ah}}{2}\left( \frac{2\pi }{z}\right) ^{1/2}+O(1), \end{aligned}$$

3.8 $$\begin{aligned} \sum _{n\ge 0}(-1)^{n}\frac{q^{m\frac{n(n+1)}{2}+mn+a}}{1-q^{mn+a}}=\frac{{\textbf {1}}_{m\mid ah}}{2z} \left( \psi \left( \frac{m+a}{2m}\right) - \psi \left( \frac{a}{2m}\right) \right) +O(|z|^{-1/2}), \end{aligned}$$

and

3.9 $$\begin{aligned} \sum _{n\ge 1}\frac{q^{an}}{1+q^{mn}}= \frac{{\textbf {1}}_{m\mid ah}}{2z}\left( \psi \left( \frac{m+a}{2m}\right) - \psi \left( \frac{a}{2m}\right) \right) +O(1). \end{aligned}$$

#### Proof

Let $z=x+\textrm{i}y$ with $x>0$ and $y\in {\mathbb {R}}$, then we have $|y|\le x\cdot \tan (\Delta )$. The proofs of (3.7) and (3.9) use similar arguments. In fact, note that

$$\begin{aligned} S(u):=\sum _{1\le n\le u}\zeta _{m}^{ahn}=u\cdot {\textbf {1}}_{m\mid ah}+O(1), \end{aligned}$$

uniformly for all $u>0$. Hence, Abel’s summation formula implies

$$\begin{aligned} \sum _{n\ge 1} &e^{-z\frac{n(n-1)}{2}-\frac{an}{m}z}\zeta _{m}^{ahn} =S(u)e^{-\frac{u(u-1)}{2}z-\frac{a}{m}uz}\big |_{u=0}^{+\infty }-\int \limits _{0}^{\infty }S(u)\left( e^{-\frac{u(u-1)}{2}z-\frac{a}{m}uz}\right) '\textrm{d}u\\&={\textbf {1}}_{m\mid ah}\cdot \int \limits _{0}^{\infty }u \left( e^{-\frac{u(u-1)}{2}z-\frac{a}{m}uz}\right) '\textrm{d}u+ O\left( \int \limits _{0}^{\infty } \left| \left( e^{-\frac{u(u-1)}{2}z-\frac{a}{m}uz}\right) '\right| \textrm{d}u\right) \\&={\textbf {1}}_{m\mid ah}\cdot \int \limits _{0}^{\infty }e^{-\frac{u(u-1)}{2}z-\frac{a}{m}uz}\textrm{d}u+ O\left( |z|\int \limits _{0}^{\infty }\left| u-\frac{1}{2}+ \frac{a}{m}\right| e^{-\frac{u(u-1)}{2}x-\frac{a}{m}ux}\textrm{d}x\right) \\&={\textbf {1}}_{m\mid ah}\cdot \int \limits _{\frac{a}{m}-\frac{1}{2}}^{\infty } e^{-\frac{u^2z}{2}+\frac{(a/m-1/2)^2}{2}z}\textrm{d}u+ O\left( |z|\int \limits _{\frac{a}{m}-\frac{1}{2}}^{\infty }|u| e^{-\frac{u^2x}{2}+\frac{(a/m-1/2)^2}{2}x}\textrm{d}u\right) \\&=\frac{{\textbf {1}}_{m\mid ah}}{2}\left( \frac{2\pi }{z}\right) ^{1/2}+O(1). \end{aligned}$$

Abel’s summation formula further implies

$$\begin{aligned} \sum _{n\ge 1} &\frac{e^{-azn/m}}{1+e^{-nz}}e^{2\pi \textrm{i}ahn/m} =S(u)\frac{e^{-azu/m}}{1+e^{-uz}}\bigg |_{u=0}^{+\infty }- \int \limits _{0}^{\infty }S(u)\left( \frac{e^{-azu/m}}{1+e^{-uz}}\right) '\textrm{d}u\\&={\textbf {1}}_{m\mid ah}\cdot \int \limits _{0}^{\infty }u \left( \frac{e^{-azu/m}}{1+e^{-uz}}\right) '\textrm{d}u+ O\left( \int \limits _{0}^{\infty }\left| \left( \frac{e^{-azu/m}}{1+e^{-uz}}\right) '\right| \textrm{d}u\right) \\&={\textbf {1}}_{m\mid ah}\cdot \int \limits _{0}^{\infty }\frac{e^{-azu/m}}{1+e^{-uz}}\textrm{d}u+O\left( |z|\int \limits _{0}^{\infty } \frac{e^{aux/m}+e^{-(1-a/m)ux}}{|e^{auz/m}+e^{-(1-a/m)uz}|^2}\textrm{d}x\right) \\&=\frac{{\textbf {1}}_{m\mid ah}}{z}\int \limits _{0}^{\infty } \sum _{k\ge 0}(-1)^{k}e^{-(k+a/m)u}\textrm{d}u+ O\left( 1+\int \limits _{1/|z|}^{\infty }\frac{|z|e^{-aux/m}\textrm{d}u}{(1-e^{-ux})^2}\right) \\&=\frac{{\textbf {1}}_{m\mid ah}}{z}\sum _{k\ge 0}\frac{(-1)^{k}}{k+a/m}+O(1). \end{aligned}$$

By using [9, Eq. 5.7.7], we have

3.10 $$\begin{aligned} \sum _{k\ge 0}\frac{(-1)^k}{k+a/m}= \frac{1}{2}\left( \psi \left( \frac{m+a}{2m}\right) - \psi \left( \frac{a}{2m}\right) \right) , \end{aligned}$$

which completes the proof of (3.9).

It remains to give the proof of (3.8). Note that

$$\begin{aligned}&\sum _{n\ge 0}(-1)^{n}\frac{q^{m\frac{n(n+1)}{2}+mn+a}}{1-q^{mn+a}}\\  &=\sum _{k\ge 0}\left( \frac{(1-q^m)q^{mk(2k+1)-a}}{(q^{-(2mk+a)}-1)(q^{-(2km+m+a)}-1)}+ \frac{(1-q^{m(2k+1)})q^{mk(2k+1)}}{q^{-(2mk+a)}-1}\right) . \end{aligned}$$

We have

$$\begin{aligned} \sum _{k\ge 0}\frac{(1-q^{m(2k+1)})q^{mk(2k+1)}}{q^{-(2mk+a)}-1}&\ll \sum _{k\ge 0}\frac{|1-e^{-(2k+1)z}|e^{-k^2x}}{e^{(2k+a/m)x}-1}\\&\ll \sum _{0\le k\le 1/|z|}\frac{(2k+1)|z|e^{-k^2x}}{(2k+a/m)x}+ \sum _{k\ge 1/|z|}e^{-k^2x}\\&\ll \sum _{k\ge 0}e^{-k^2x}\ll x^{-1/2}\ll |z|^{-1/2}, \end{aligned}$$

and

$$\begin{aligned}&\sum _{k\ge 0}\frac{(1-q^m)q^{mk(2k+1)-a}}{(q^{-(2mk+a)}-1)(q^{-(2km+m+a)}-1)}\\&= \sum _{0\le k\le |z|^{-1/2}}\frac{(1-e^{-z})e^{-z(k(2k+1)-a/m)}\zeta _m^{-ah}}{(e^{(2k+a/m)z}\zeta _{m}^{ah}-1)(e^{(2k+1+a/m)z}\zeta _{m}^{ah}-1)}\\&\qquad +O\left( \sum _{k\ge |z|^{-1/2}}\frac{|z|e^{-k^2x}}{(2k+a/m)(2k+1+a/m)x^2}\right) . \end{aligned}$$

For the *O*-term, we estimate that

$$\begin{aligned} \sum _{k\ge |z|^{-1/2}}\frac{|z|e^{-k^2x}}{(2k+a/m)(2k+1+a/m)x^2}\ll \frac{1}{|z|} \sum _{k\ge |z|^{-1/2}}\frac{1}{k^2}\ll |z|^{-1+1/2}\ll |z|^{-1/2}. \end{aligned}$$

We now estimate the main term. Since $0\le k\le |z|^{-1/2}$, for $m\not \mid ah$, we have

$$\begin{aligned} \sum _{0\le k\le |z|^{-1/2}}\frac{(1-e^{-z})e^{-z(k(2k+1)-a/m)}\zeta _m^{-ah}}{(e^{(2k+a/m)z}\zeta _{m}^{ah}-1)(e^{(2k+1+a/m)z}\zeta _{m}^{ah}-1)}&\\\ll \sum _{0\le k\le |z|^{-1/2}}\frac{|z|}{\left| |\zeta _{m}^{ah}-1|+ O(z^{1/2})\right| ^2}\ll |z|^{1/2}&. \end{aligned}$$

For $m\mid ah$, we have

$$\begin{aligned}&\sum _{0\le k\le |z|^{-1/2}}\frac{(1-e^{-z})e^{-z(k(2k+1)-a/m)}\zeta _m^{-ah}}{(e^{(2k+a/m)z}\zeta _{m}^{ah}-1)(e^{(2k+1+a/m)z}\zeta _{m}^{ah}-1)}\\&=\sum _{0\le k\le |z|^{-1/2}}\frac{z}{(2k+a/m)(2k+1+a/m)z^2} (1+O((k+1)^2z))(1+O((k+1)z))\\&=\frac{1}{z}\sum _{0\le k\le |z|^{-1/2}}\frac{1}{(2k+a/m)(2k+1+a/m)}+ O\left( \sum _{0\le k\le |z|^{-1/2}}1\right) \\&=\frac{1}{z}\sum _{k\ge 0} \frac{(-1)^k}{k+a/m}+O(|z|^{-1/2}). \end{aligned}$$

Combining the above estimates, we get

$$\begin{aligned} \sum _{n\ge 0}(-1)^{n}\frac{q^{m\frac{n(n+1)}{2}+mn+a}}{1-q^{mn+a}}= \frac{{\textbf {1}}_{m\mid ah}}{2z}\left( \psi \left( \frac{m+a}{2m}\right) - \psi \left( \frac{a}{2m}\right) \right) +O(|z|^{-1/2}), \end{aligned}$$

which completes the proof of the lemma. $\square$

#### Lemma 3.4

We have

$$\begin{aligned} G_{01}\!\left( a,m;e^{-z/m}\right) = \frac{1+O(|z|^{1/2})}{2} \frac{1}{\sqrt{2}}\exp \left( \frac{\pi ^2m}{12z}\right) , \\ G_{10}\!\left( a,m;e^{-z/m}\right) =(1+O(|z|^{1/2})) \frac{\psi \left( \frac{m+a}{2m}\right) - \psi \left( \frac{a}{2m}\right) }{2\pi \csc (a\pi /m)} \left( \frac{z}{2\pi m}\right) ^{1/2}\exp \left( \frac{\pi ^2m}{6z}\right) , \end{aligned}$$

and

$$\begin{aligned} G_{11}\!\left( a,m;e^{-z/m}\right) =(1+O(|z|^{1/2})) \frac{\psi \left( \frac{m+a}{2m}\right) - \psi \left( \frac{a}{2m}\right) }{2\pi \csc (a\pi /m)} \left( \frac{z}{4\pi m}\right) ^{1/2}\exp \left( \frac{\pi ^2m}{4z}\right) \end{aligned}$$

when $z\rightarrow 0$ in every fixed Stolz angle $|\arg (z)|\le \Delta$ $(0<\Delta <\pi /2)$.

#### Proof

This proof of the lemma can be straightforwardly done using Proposition 3.2 and Lemma 3.3; we omit the details. $\square$

#### Lemma 3.5

For $(x,y)\in S$, we have

$$\begin{aligned} G_{xy}\!\left( a,m; e^{-z/m}\zeta _{m}^h\right) = \left( {\textbf {1}}_{m\mid h}+O(|z|^{1/2})\right) G_{xy}\!\left( a,m;e^{-z/m}\right) , \end{aligned}$$

when $z\rightarrow 0$ in every fixed Stolz angle $|\arg (z)|\le \Delta$ $(0<\Delta <\pi /2)$.

#### Proof

This proof of the lemma can be straightforwardly done using Proposition 3.2, Lemmas 3.3 and 3.4. We sketch some of the details. For $q=e^{-z/m}\zeta _{m}^h$ with $m\not \mid h$, we have

$$\begin{aligned}&G_{01}\!\left( a,m;e^{-z/m}\zeta _{m}^h\right) \\&=\frac{(e^{-2z/m}\zeta _m^{2h};e^{-2z/m}\zeta _m^{2h})_\infty }{(e^{-z/m}\zeta _m^{h};e^{-z/m}\zeta _m^{h})_\infty } \frac{\sum _{n\ge 1}q^{m\frac{n(n-1)}{2}+an}}{(e^{-az/m}\zeta _{2m}^{m+2ah},e^{-(1-a/m)z}\zeta _{2m}^{-m-2ah}, e^{-z};e^{-z})_\infty }\\&\ll \left( |z|^{1/2}+{\textbf {1}}_{m\mid ah}\right) \exp \left( \frac{\gcd (m,h)^2}{6mz/\pi ^2}+\frac{\pi ^2}{6z}+ \frac{B_2\left( \left\{ \frac{2ah+m}{2m}\right\} \right) }{z/(2\pi ^2)}- \frac{\gcd (m,2h)^2}{12mz/\pi ^2}\right) \\&\ll \left( |z|^{1/2}+{\textbf {1}}_{m\mid ah}\right) G_{01}\!\left( a,m;e^{-z/m}\right) \exp \left( -\frac{2\pi ^2}{z} A_{01}(a,m; h)\right) , \end{aligned}$$

where

$$\begin{aligned} A_{01}(a,m; h)=\frac{m}{24}+\frac{\gcd (m,2h)^2}{24m}- \frac{\gcd (m,h)^2}{12m}-\frac{1}{12}- B_2\left( \left\{ \frac{2ah+m}{2m}\right\} \right) . \end{aligned}$$

Similarly, we have

$$\begin{aligned}&G_{10}\!\left( a,m;e^{-z/m}\zeta _{m}^h\right) \\&=\frac{(\zeta _{m}^{ah}e^{-az/m},\zeta _{m}^{-ah}e^{-(1-a/m)z};e^{-z})_\infty }{(e^{-z};e^{-z})_\infty ^2( e^{-z/m}\zeta _m^{h};e^{-z/m}\zeta _m^h)_\infty } \sum _{k\ge 0}(-1)^{k}\frac{q^{m\frac{k(k+1)}{2}+mk+a}}{1-q^{mk+a}}\\&\ll \left( 1+|z|^{-1/2}  {\textbf {1}}_{m\mid ah}\right) |z|\exp \left( \frac{\pi ^2}{3z}+\frac{\pi ^2\gcd (m,h)^2}{6mz}- \frac{2\pi ^2}{z}B_2\left( \left\{ \frac{a h}{m}\right\} \right) \right) \\&\ll \left( |z|^{1/2}+{\textbf {1}}_{m\mid ah}\right) G_{10}\!\left( a,m;e^{-z/m}\right) \exp \left( -\frac{2\pi ^2}{z} A_{10}(a,m; h)\right) , \end{aligned}$$

where

$$\begin{aligned} A_{10}(a,m; h)=\frac{m}{12}+B_2\left( \left\{ \frac{a h}{m}\right\} \right) - \frac{1}{6}-\frac{\gcd (m,h)^2}{12m}; \end{aligned}$$

and

$$\begin{aligned}&G_{11}\!\left( a,m;e^{-z/m}\zeta _{m}^h\right) \\&=2\frac{(e^{-2z/m}\zeta _{m}^{2h};e^{-2z/m}\zeta _{m}^{2h})_\infty (e^{-2z};e^{-2z})_\infty ^2}{(e^{-z/m}\zeta _{m}^{h};e^{-z/m}\zeta _{m}^{h})_\infty ^2 (e^{-z};e^{-z})_\infty ^4}\\&\quad \times \frac{(e^{-az/m}\zeta _{m}^{ah},e^{-(1-a/m)z} \zeta _{m}^{-ah};e^{-z})_\infty }{(e^{-az/m}\zeta _{2m}^{m+2ah},e^{-(1-a/m)z}\zeta _{2m}^{-m-2ah}; e^{-z})_\infty }\sum _{n\ge 1}\frac{q^{an}}{1+q^{mn}}\\&\ll \left( |z|+{\textbf {1}}_{m\mid ah}\right) |z|^{1/2} \exp \left( \frac{\pi ^2\gcd (m,h)^2}{3mz}- \frac{\pi ^2\gcd (m,2h)^2}{12mz}+\frac{\pi ^2}{2z}\right) \\&\quad \times \exp \left( -\frac{2\pi ^2}{z} \left( B_2\left( \left\{ \frac{a h}{m}\right\} \right) - B_2\left( \left\{ \frac{2ah+m}{2m}\right\} \right) \right) \right) \\&\ll \left( |z|+{\textbf {1}}_{m\mid ah}\right) G_{11}\!\left( a,m;e^{-z/m}\right) \exp \left( -\frac{2\pi ^2}{z} A_{11}(a,m;h)\right) , \end{aligned}$$

where

$$\begin{aligned} A_{11}(a,m;h)=\frac{m}{8}+\frac{\gcd (m,2h)^2}{24m}+ B_2\left( \left\{ \frac{a h}{m}\right\} \right) - \frac{1}{4}-\frac{\gcd (m,h)^2}{6m}- B_2\left( \left\{ \frac{2ah+m}{2m}\right\} \right) . \end{aligned}$$

It is clear that $A_{11}(a,m;h)=A_{01}(a,m;h)+A_{10}(a,m;h)$. Using elementary arguments, one can show that $A_{xy}(a,m;h)$ with $(x,y)\in S$, is nonnegative for all integers $m\not \mid h$ and $m\ge 3$. Moreover, for $m\mid ah$ but $m\not \mid h$, we have

$$\begin{aligned} A_{01}(a,m; h)=\frac{m}{24}+\frac{\gcd (m,2h)^2}{24m}- \frac{\gcd (m,h)^2}{12m}\ge \frac{m}{24}-\frac{\gcd (m,h)^2}{24m}\ge \left( 1-\frac{1}{4}\right) \frac{m}{24}>0, \end{aligned}$$

and

$$\begin{aligned} A_{10}(a,m; h)=\frac{m}{12}-\frac{\gcd (m,h)^2}{12m}\ge \left( 1-\frac{1}{4}\right) \frac{m}{12}>0, \end{aligned}$$

because of $\gcd (m,2h)\ge \gcd (m,h)$ and $\gcd (m,h)\le m/2$. The combination of all the above estimates proves the lemma. $\square$

### Proof of Theorems 1.3 and 1.4

In this subsection, we will provide the proof of Theorem 1.3. To accomplish this, we will first establish Theorem 1.4, the residue class analogue of Ingham’s Tauberian theorem. A special case of Ingham’s Tauberian theorem, as presented in [14, p.1082, Eqs.(21),(22)], is stated as the following theorem.

#### Theorem 3.6

Let *A*(*u*) be an increasing function on $[0, \infty )$ with $A(0)=0$ be defined as the following Laplace transform:

3.11 $$\begin{aligned} f(s)=\int \limits _{0}^{\infty }e^{-us}\textrm{d}A(u). \end{aligned}$$

If $f(s)\sim f_0(s)$ when $s\rightarrow 0$ in every fixed Stolz angle $|\arg (s)|\le \Delta$ $(0<\Delta <\pi /2)$, where

$$\begin{aligned} f_0(s)=C(M/s)^{v\beta -\frac{1}{2}}e^{\beta ^{-1}(M/s)^{\beta }}\quad (\beta , M, C>0, v\in {\mathbb {R}}). \end{aligned}$$

Then with $\alpha =\beta /(1+\beta )$, we have:

$$\begin{aligned} A(\omega )\sim \left( \frac{1-\alpha }{2\pi }\right) ^{\frac{1}{2}} C(M\omega )^{v\alpha -\frac{1}{2}}e^{\alpha ^{-1}(M\omega )^{\alpha }}, \text { as }\omega \rightarrow +\infty . \end{aligned}$$

Let $F(q)=\sum _{n\ge 0}c_nq^n$ be a power series whose coefficients $c_n$ are nonnegative and increasing, and converge absolutely for $|q|<1$. Notice that (3.11) involves a Riemann–Stieltjes integral. Therefore, if we define

$$\begin{aligned} A(u)=c_{\lfloor u\rfloor }-c_{0}, \end{aligned}$$

for any $u\ge 0$, then *A*(*u*) is nonnegative and increasing with $A(0)=0$. Integration by parts for a Riemann–Stieltjes integral yields

3.12 $$\begin{aligned} \int \limits _{0}^{\infty }e^{-us}\textrm{d}A(u)=s\int \limits _{0}^{\infty }A(u)e^{-us}\textrm{d}u= \sum _{k\ge 0}A(k)\int \limits _{k}^{k+1}s e^{-us}\textrm{d}u&\nonumber \\ =(1-e^{-s})F(e^{-s})-c_0&. \end{aligned}$$

As a conclusion, if $F(e^{-s})$ meets the conditions in Theorem 3.6, then we can use that theorem to derive an asymptotic formula for $c_n$.

However, in our case it is not easy to prove that $p_n(a,m-a,m;x,y)$ is increasing for $n\ge 0$. We only have

3.13 $$\begin{aligned} p_{n+m}(a,m-a,m;x,y)\ge p_{n}(a,m-a,m;x,y), \end{aligned}$$

for all $n\ge 0$, due to Lemma 2.1. Luckily, we can prove Theorem 1.4, a residue class analogue of Ingham’s Tauberian theorem, which works in our case.

#### Proof of Theorem 1.4

Due to condition (1) of Theorem 1.4, $c_{mn+j}$ is increasing in *n*, for any $0\le j<m$. Moreover, we have

$$\begin{aligned} \sum _{n\ge 0}c_{mn+j}q^{n}=\frac{1}{m}\sum _{0\le h<m}e^{-2\pi \textrm{i}hj}q^{-j/m} f\!\left( q^{1/m}e^{2\pi \textrm{i}h/m}\right) , \end{aligned}$$

by using the orthogonality of the *m*-th roots of unity. Using condition (2) of Theorem 1.4 and the above, with $A_{j}(u)=c_{m\lfloor u\rfloor +j}-c_j$, we have

$$\begin{aligned} c_j+\int \limits _{0}^{\infty }e^{-uz}\textrm{d}A_{j}(u)= (1-e^{-z})\sum _{n\ge 0}c_{mn+j}e^{-nz}\sim \frac{\alpha }{m^{1+\gamma }} z^{1+\gamma }e^{m^\rho \beta z^{-\rho }/\rho }, \end{aligned}$$

by using (3.12), when $z\rightarrow 0$ in every fixed Stolz angle $|\arg (z)|\le \Delta$, $(0<\Delta <\pi /2)$. Hence, Theorem 3.6 implies

$$\begin{aligned} c_{mn+j}\sim \frac{\alpha \beta ^{\frac{1+2\gamma }{2(1+\rho )}}}{\sqrt{2\pi (1+\rho )}}(mn)^{-\frac{1+2\gamma }{2(1+\rho )}- \frac{1}{2}}e^{(1+1/\rho )\beta ^{1/(1+\rho )} (mn)^{\rho /(1+\rho )}}, \end{aligned}$$

as $n\rightarrow +\infty$. Therefore, by using the simple estimates

$$\begin{aligned} (n+r)^{\kappa _1}\sim n^{\kappa _1}\quad \text {and}\quad (n+r)^{\kappa _2}=n^{\kappa _2}+O(n^{\kappa _2-1})=n^{\kappa _2}+o(1), \end{aligned}$$

as $n\rightarrow \infty$, for any given $\kappa _1\in {\mathbb {R}}$ and $\kappa _2<1$, we can replace *mn* by $nm+j$ in above asymptotic formula of $c_f(mn+j)$. This completes the proof of Theorem 1.4. $\square$

We now give the proof of Theorem 1.3. From inequality (3.13), Lemmas 3.4 and 3.5, we see that the generating function $G_{xy}(a,m;q)$ with $(x,y)\in S$ meets the conditions of Theorem 1.4. Thus, we have

$$\begin{aligned} p_{n}(a,m-a,m; x,y)\sim c_{a,m}(x){\widehat{p}}_n(x,y), \end{aligned}$$

as $$n\rightarrow +\infty $$, with

$$\begin{aligned} \left( c_{a,m}(0), c_{a,m}(1)\right) = \left( \frac{1}{2},~\frac{\psi \left( 1/2+a/2m\right) - \psi \left( {a}/{2m}\right) }{2\pi \csc (a\pi /m)}\right) . \end{aligned}$$

Here,

$$\begin{aligned} {\widehat{p}}_n(0,1)=\frac{e^{\pi \sqrt{n/3}}}{4\root 4 \of {3}n^{3/4}},\;\; {\widehat{p}}_n(1,0)= \frac{e^{2\pi \sqrt{n/6}}}{4\sqrt{3}n},\;\; \text {and}\;\; {\widehat{p}}_n(1,1)= \frac{1}{8n}e^{\pi \sqrt{n}}. \end{aligned}$$

Recall the unrestricted partition function *p*(*n*) (see [13, Eq. (1.41)]), the distinct partition function *q*(*n*) (see [13, pages 109–110]), and the overpartition function ${\bar{p}}(n)$ (see [14, Eq. (1)]), which have the following asymptotic formulas:

$$\begin{aligned} q(n)\sim \frac{e^{\pi \sqrt{n/3}}}{4\root 4 \of {3}n^{3/4}}, \;\; p(n)\sim \frac{e^{2\pi \sqrt{n/6}}}{4\sqrt{3}n}, \;\; \text {and} \;\; {\bar{p}}(n)\sim \frac{1}{8n}e^{\pi \sqrt{n}}, \end{aligned}$$

as $n\rightarrow \infty$. This completes the proof of Theorem 1.3.

## Remarks on residue class biases in distinct partitions

Theorem 1.1 provides results for biases in residue classes in partitions and overpartitions. For distinct partitions, we only have a partial result, given in Theorem 1.2. Beyond that partial result, we provide a conjecture, proposed in the end of this section.

In the following, we write $d_n(a,b;m):=p_n(a,b,m; 0,1)$ for brevity. Then,

$$\begin{aligned} \sum _{n\ge 0}d_n(a,b;m)q^n= \sum _{\begin{array}{c} \lambda \in {{\mathcal {D}}}\\ \ell _{a,m}(\lambda )> \ell _{b,m}(\lambda ) \end{array}} q^{|\lambda |}. \end{aligned}$$

If $b=m-a$, then Lemma 3.1 implies

4.1 $$\begin{aligned} \sum _{n\ge 0}d_n(a,m-a;m)q^{n}=\frac{(-q;q)_\infty }{(-q^a,-q^{m-a},q^m;q^m)_\infty }\sum _{n\ge 1}q^{m\left( {\begin{array}{c}n\\ 2\end{array}}\right) +n a}. \end{aligned}$$

From Equation (4.1), we have the following inequalities for $d_{n}(1,2;3)$ and $d_{n}(2,1;3)$.

### Proposition 4.1

We have

$$\begin{aligned} d_{3n+2}(1,2;3)\le d_{3n+2}(2,1;3), \\ d_{3n}(1,2;3)\ge d_{3n}(2,1;3), \quad \text {and}\quad d_{3n+1}(1,2;3)\ge d_{3n+1}(2,1;3), \end{aligned}$$

for all $$n\ge 0$$.

### Proof

The proof is straightforward and uses a dissection strategy. First, we observe

$$\begin{aligned} \sum _{n\ge 0}(d_{n}(1,2;3)-d_{n}(2,1;3))q^n= \frac{(-q^3;q^3)_\infty }{(q^3;q^3)_\infty } \sum _{n\ge 1}q^{\frac{n(3n-1)}{2}}(1-q^{n}). \end{aligned}$$

Dissection of the last sum gives

$$\begin{aligned}&\sum _{n\ge 0}q^{\frac{n(3n-1)}{2}}(1-q^{n}) =\sum _{0\le j\le 2}\sum _{n\ge 0}q^{\frac{3(3n+j)-3n+j)}{2}}(1-q^{3n+j})\\&=\sum _{n\ge 0}q^{\frac{3n(9n-1)}{2}}(1-q^{3n})+ q\sum _{n\ge 0}q^{\frac{9n(3n+5)}{2}}(1-q^{12n+6})- q^2\sum _{n\ge 0}q^{\frac{9n(3n+7)}{2}}(1-q^{6n+3}). \end{aligned}$$

This completes the proof. $\square$

A similar argument can be applied to compare $d_{n}(1,3;4)$ and $d_{n}(3,1;4)$:

$$\begin{aligned} \sum _{n\ge 0}\left( d_n(1,3;4)-d_n(3,1;4)\right) q^{n} =\frac{(-q;q)_\infty }{(-q,-q^{3},q^4;q^4)_\infty } \sum _{n\ge 1}q^{2n^2-n}(1-q^{n})&\\ =\frac{1}{(q^2;q^2)_\infty }\sum _{n\ge 1}q^{2n^2-n}(1-q^{2n}) =\frac{1}{(q^4;q^2)_\infty }\sum _{n\ge 1}q^{2n^2}\sum _{0\le j<n}q^{2j-n}&. \end{aligned}$$

Therefore, $d_n(1,3;4)\ge d_n(3,1;4)$. This is also a special case of Theorem 1.2.

We turn to the general case. For any $1\le a<b\le m$, by Equation (2.1) we have

$$\begin{aligned} \sum _{n\ge 0}d_{n}(a,b;m)q^n= \frac{(-q;q)_\infty }{(-q^a,-q^b;q^m)_\infty } \sum _{\begin{array}{c} j> k\\ j,k\ge 0 \end{array}}\frac{q^{\frac{1}{2}m(j(j-1)+k(k-1))+aj+bk}}{(q^m;q^m)_{j}(q^m;q^m)_{k}}. \end{aligned}$$

Based on this generating function and extensive computer experiments, we propose the following conjecture.

### Conjecture 4.1

For any positive integers *a*, *b*, *m* such that $1\le a<b\le m$, $m\ge 3$ and $(a,b,m)\ne (1,2,3)$, there exists a constant $N_{a,b,m}>0$ such that

$$\begin{aligned} d_{n}(a,b; m)\ge d_{n}(b,a; m),\quad \text {for all}\quad n\ge N_{a,b, m}. \end{aligned}$$

Moreover,

$$\begin{aligned} N_{a,m-a,m}=0,\quad \text {for all}\quad 1\le a<m/2, \end{aligned}$$

except

$$\begin{aligned} N_{2,3,5}=45, N_{2,4,6}=5, N_{3,4,7}=8, N_{4,5,9}=9. \end{aligned}$$

### Remark 4.1

The constants $N_{a,b, m}$ appear to be all relatively small. In particular, there exists an $m_0>9$ such that $N_{a,b, m}$ equals zero for all $m\ge m_0$.

### Remark 4.2

We note that we confirmed Conjecture 4.1 for a class of special cases in Theorem 1.2.

## Data Availability

Data sharing is not applicable to this article.

## Footnotes

1 Here, we used the fact that

$$\begin{aligned} \frac{1}{2}\left( \zeta _\mu (2)+\zeta _{1-\mu }(2)\right) = \sum _{n\ge 1}\frac{e^{2\pi \textrm{i}\mu n}+e^{2\pi \textrm{i}(1-\mu ) n}}{2n^2}= \sum _{n\ge 1}\frac{\cos (2\pi n \mu )}{n^2}=\pi ^2B_2(\mu ), \end{aligned}$$

which can be found in [9, Eq. 25.12.8] where $\zeta _\mu (s):=\sum _{n\ge 1}\frac{e^{2\pi \textrm{i}\mu n}}{n^s}$ is the periodic zeta function.
